# Analysis of the composition and function of rhizosphere microbial communities in plants with tobacco bacterial wilt disease and healthy plants

**DOI:** 10.1128/spectrum.00559-24

**Published:** 2024-10-29

**Authors:** Bingye Yang, Chaoqun Zhang, Chengwei Guan, Xiaohu Feng, Ding Yan, Zhigao Zhang, Yanmin Qin, Shubin Xiong, Wenmei Zhang, Xianjie Cai, Liwei Hu

**Affiliations:** 1 Zhengzhou Tobacco Research Institute of CNTC, Zhengzhou, China; 2 Jiangxi Tobacco Science Institute, Nanchang, China; 3 Fuzhou Branch of Jiangxi Provincial Tobacco Company, Fuzhou, Jiangxi, China; 4 Procurement Center, Shanghai Tobacco Group Co., Ltd., Shanghai, China; Institute of Microbiology, Chinese Academy of Sciences, Beijing, China

**Keywords:** tobacco bacterial wilt, physicochemical properties, rhizosphere microbial community composition and function, metagenomic

## Abstract

To explore the factors influencing the occurrence of bacterial wilt, the differences in the physicochemical properties, microbial community composition and function between rhizosphere soil of tobacco plants with bacterial wilt and healthy plants in the tobacco planting area of Fuzhou City, Jiangxi Province were analyzed and compared. The results showed that the rhizosphere soil of diseased tobacco exhibited significantly reduced levels of exchangeable potassium, water-soluble potassium, nitrate nitrogen, total nitrogen and pH, in comparison to the rhizosphere soil of healthy plants. Conversely, the available phosphorus content of the rhizosphere soil of diseased tobacco was significantly increased. The amount of *Ralstonia solanacearum* in soil was negatively correlated with pH, nitrate nitrogen and total nitrogen, and positively correlated with exchangeable potassium and water-soluble potassium. A total of 43 genera were significantly different between the two groups of rhizosphere soil, of which 24 genera were enriched in the rhizosphere of healthy plants, including *Ideonella*, *Rhizophagus*, *Rhizobacter*, *Altererythrobacter* and *Ignavibacterium* associated with plant disease resistance, *Thermodesulfovibrio*, *Syntrophorhabdus*, *Syntrophus*, *Chlorobium*, *Hydrogenophaga* and *Limnohabitans* associated with soil sulfur metabolism, as well as *Ignavibacterium*, *Ideonella*, *Derxia* and *Azohydromonas* associated with soil nitrogen cycling. Kyoto Encyclopedia of Genes and Genomes functional analysis of the unigenes obtained by metagenomic sequencing also showed that the differential unigenes were significantly enriched in the sulfur metabolism pathway. In addition, the rhizosphere soil of diseased tobacco plants exhibited a higher abundance of antibiotic-producing actinomycetes and an increased load of antibiotic resistance genes compared to that of healthy plants. In general, lower pH value, less content of nitrate nitrogen and total nitrogen, and more content of exchangeable potassium and water-soluble potassium could contribute to onset of bacterial wilt. Twenty-four genera, including *Ideonella* and *Rhizophagus*, may construct a healthy microecological network in the rhizosphere of tobacco plants. All these factors may interact with each other to control the development of bacterial wilt. This complicated interaction network needs to be explored further.

**IMPORTANCE:**

Previous studies have mainly focused on the differences in microbial species composition between healthy and diseased soils, but the differences in microbial community functions between two types of soil have not been well characterized. In this study, soil samples in diseased and healthy plant rhizospheres were collected for physicochemical property testing and metagenomic sequencing. We focused on analyzing the differences in physicochemical properties and microbial community functions between these soils, as well as the correlation between these factors and pathogen content. The results of this study provide a theoretical basis for further understanding the occurrence of tobacco bacterial wilt in the field.

## INTRODUCTION

Due to its high economic value, continuous cropping of tobacco is very common, leading to the accumulation of pathogens in the soil and serious soil-borne diseases. Tobacco bacterial wilt is a devastating soil-borne disease caused by *Ralstonia solanacearum*, which is distributed in almost all tobacco plant-growing areas around the world (1). Pathogens can survive up to 40 years in soil and infected plant sites, and can be transmitted from plant to plant through irrigation water, agricultural operations, and nematodes (2). After being infected by *R. solanacearum*, the tobacco leaves will wilt and turn yellow on the side that has the vasculature impaired, and eventually, the whole plant will die. Moreover, there will be many green and variegated leaves after curing, accompanied by an uncoordinated chemical composition, which will have a huge impact on the quality of the tobacco products (3). The average incidence rate of tobacco bacterial wilt in 22 main tobacco-growing regions in China is about 15%–35% but can reach up to 75% in high-temperature and high-humidity areas with suitable climate, which has become an important factor restricting tobacco production (4).

The bacterial wilt pathogen, *R. solanacearum*, has been shown to significantly affect the soil physicochemical properties of tobacco plants (5). Specifically, there were significant alterations in soil pH, available nitrogen, available potassium and available phosphorus in tobacco-planted soils with and without *R. solanacearum* inoculation (5, 6). Additionally, the invasion of *R. solanacearum* in other Solanaceae crops also resulted in changes to soil physicochemical properties (7, 8). Furthermore, *R. solanacearum* infection changed the composition of soil microorganisms in the rhizosphere, significantly reducing the abundance of actinomycetes and proteobacteria while increasing the abundance of soil-borne pathogen *Fusarium solani* (9). Metagenomic sequencing analysis showed that the load of microbial nitrogen metabolism genes in rhizosphere soil changed significantly after *R. solanacearum* infection, resulting in a stronger denitrification process in soil (10).

Microorganisms occupy a core position within soil biota and make crucial contributions to soil ecosystem processes, nutrient cycling, and sustainable development of resources and the environment (11). In addition, soil microorganisms also have a crucial impact on plant life activities, which can promote plant growth and development, enhance plant nutrient absorption capacity, and improve plant resistance to biotic and abiotic stresses, thereby contributing to increased crop yields (12–14). In recent years, with the development of metagenomics technology, more and more evidence has shown that soil microbial communities are associated with the severity of soil-borne diseases. For example, Zhang and his colleagues performed a correlation network analysis of bacterial populations in tomato bacterial wilt-suppressive soil and conducive soil, and the results showed that there were more interactions among bacterial populations in the resistant soil samples, and a more stable network with more complex interactions was constructed, which was conducive to inhibiting the growth of pathogens (15). Therefore, analyzing the composition and function of microorganisms in infected and healthy soils is of great significance for understanding the occurrence of tobacco wilt disease.

Soil physicochemical properties can drive microbial composition, inducing general or specific disease-suppressive soil, and then affect plant health (16). Based on the different effects of *R. solanacearum* on the hosts in different soils, soils can be divided into conductive and inhibitory soils. According to reports, soil texture (17), organic matter content (18), carbon:nitrogen ratio (19), available phosphorus (14), electrical conductivity value (20), pH value (21) and available manganese (22) were associated with soil microbiota composition and also had close relationships with disease index. Therefore, analyzing the relationship between soil physicochemical properties and rhizosphere microbiome is conducive to linking the host, rhizosphere microorganisms and the environment.

Previous studies have shown significant changes in the microbial community structure of soil affected by bacterial wilt compared to healthy soil (5). However, the impact of alterations in microbial community structure on soil nutrient cycling and elemental decomposition ability remains relatively understudied. In this study, we hypothesized that the occurrence of bacterial wilt was due to the disruption of the rhizosphere microecological balance, including changes in soil physical, chemical and biological characteristics. So, we collected soil samples from both diseased and non-diseased plant rhizospheres and conducted physicochemical property testing and metagenomic sequencing. Our primary focus was to analyze differences in physicochemical properties and microbial community functions among different soil samples, as well as examine the correlation between these factors and pathogen content. The results revealed significant differences in particle size, exchangeable potassium, water-soluble potassium, nitrate nitrogen, total nitrogen and pH levels between the rhizosphere soil of diseased and healthy plants. Additionally, the abundance of microorganisms associated with disease resistance and soil sulfur cycling was found to be higher in the rhizosphere of healthy plants than in diseased plants. This study revealed the differences in the rhizosphere microecological environment between healthy and diseased plants and provided a new theoretical hint for the prevention and control of tobacco bacterial wilt.

## RESULTS

### Comparison of rhizosphere soil chemical composition and particle size between diseased and healthy plants

As shown in Table 1, there were extremely significant differences (*P* < 0.01) in the exchangeable potassium, water-soluble potassium and pH between the rhizosphere soil of diseased and healthy tobacco plants. Specifically, the exchangeable potassium, water-soluble potassium and pH in the rhizosphere soil of diseased plants were relatively lower, which were 152.33 mg/kg, 25.13 mg/kg and 5.27, respectively. In the rhizosphere soil of healthy plants, these indices were 237.00 mg/kg, 41.33 mg/kg and 6.1, respectively. There were significant differences (*P* < 0.05) in nitrate nitrogen, total nitrogen and available phosphorus between the rhizosphere soil of diseased and healthy tobacco plants. Among them, the contents of nitrate and total nitrogen in the rhizosphere soil of diseased plants were lower than those in healthy plants, and the content of available phosphorus in the rhizosphere soil of diseased plants was higher. There were no significant differences in ammonium nitrogen, cation exchange capacity, chloride ions, organic matter and hydrolyzed nitrogen between the rhizosphere soil of diseased and healthy tobacco plants.

**TABLE 1:** Comparison of chemical compositions of rhizosphere soils of diseased and healthy plants^*a*^

Chemical composition	Rhizosphere soil of healthy plants					Rhizosphere soil of diseased plants					
	HS1 #	HS2 #	HS3 #	Average	SD	DS1 #	DS2 #	DS3 #	Average	SD	*P* value
Nitrate nitrogen (mg/kg)	25.09	21.90	23.30	23.43	1.60	20.30	16.10	18.20	18.20	2.10	<0.05
Ammonium nitrogen (mg/kg)	14.70	10.30	22.80	15.93	6.34	13.40	21.90	20.60	18.63	4.58	>0.05
Available phosphorus (mg/kg)	63.40	47.70	42.60	51.23	10.84	84.10	73.40	64.40	73.97	9.86	<0.05
Cation exchange capacity（cmol/kg）	6.40	7.50	5.40	6.43	1.05	5.50	6.60	4.70	5.60	0.95	>0.05
Chloride ions (mg/kg)	74.10	81.20	103.00	86.10	15.06	67.30	56.60	80.4	68.10	11.92	>0.05
Exchangeable potassium (mg/kg)	175.00	135.00	147.00	152.33	20.53	238.00	238.00	235.00	237.00	1.73	<0.01
Water-soluble potassium (mg/kg)	25.10	24.50	25.80	25.13	0.65	46.70	33.60	43.70	41.33	6.86	<0.01
pH	6.30	6.00	6.00	6.10	0.17	5.10	5.10	5.60	5.27	0.29	<0.01
Organic matter (g/kg)	31.90	18.10	22.60	24.20	7.04	21.90	20.50	18.50	20.30	1.71	>0.05
Total nitrogen (%)	0.167	0.17	0.13	0.16	0.02	0.123	0.113	0.117	0.12	0.01	<0.05
Hydrolyzed nitrogen (mg/kg)	157.00	96.50	135.00	129.50	30.62	121.00	115.00	120.00	118.67	3.21	>0.05

^*a*^ Each soil sample consisted of thoroughly mixed rhizosphere soils from 15 healthy or diseased plants. The *P* value was obtained using Student's *t*-test.

We sieved the rhizosphere soil into three particle size classes: (i) <0.5-mm size (**clay**), (ii) 0.005- to 0.05-mm size (**silt**) and (iii) 0.05- to 1.0-mm size (**sand**), and found that the 0.05- to 1.0-mm particle size were the dominant fractions in the rhizosphere soil (Table 2). As shown in Table 2, there is a significant difference in the proportion of particle sizes 0.005–0.05 mm and 0.05–1.0 mm in the rhizosphere soil of diseased and healthy plants. The proportion of particles sizes 0.005–0.05 mm in the rhizosphere soil of healthy tobacco plants was 36.88% (average value), while the proportion of diseased tobacco plants was 39.14%. The proportion of particles sizes 0.005–0.05 mm in the rhizosphere soil of healthy tobacco was 62.00% (average value), while the proportion of diseased tobacco was 59.48%.

**TABLE 2:** Particle size analysis of rhizosphere soils of diseased and healthy plants^*a*^

Size classes	Rhizosphere soil of healthy plants (%)					Rhizosphere soil of diseased plants (%)					*P* value
	HS1 #	HS2 #	HS3 #	Average	SD (%)	DS1 #	DS2 #	DS3 #	Average	SD (%)	
<0.005 mm	2.81	3.64	3.09	3.18	0.42	4.00	4.04	3.96	4.00	0.04	>0.05
0.005–0.05 mm	35.61	38.72	36.31	36.88	1.63	38.71	39.21	39.51	39.14	0.40	<0.05
0.05–1.0 mm	63.39	60.01	62.61	62.00	1.77	59.92	59.40	59.12	59.48	0.41	<0.05

^*a*^ Each soil sample consisted of thoroughly mixed rhizosphere soil of 15 healthy or diseased plants. The *P* value was obtained using Student's *t*-test.

### Overview of rhizosphere soil microbial communities

**Fig 1:**
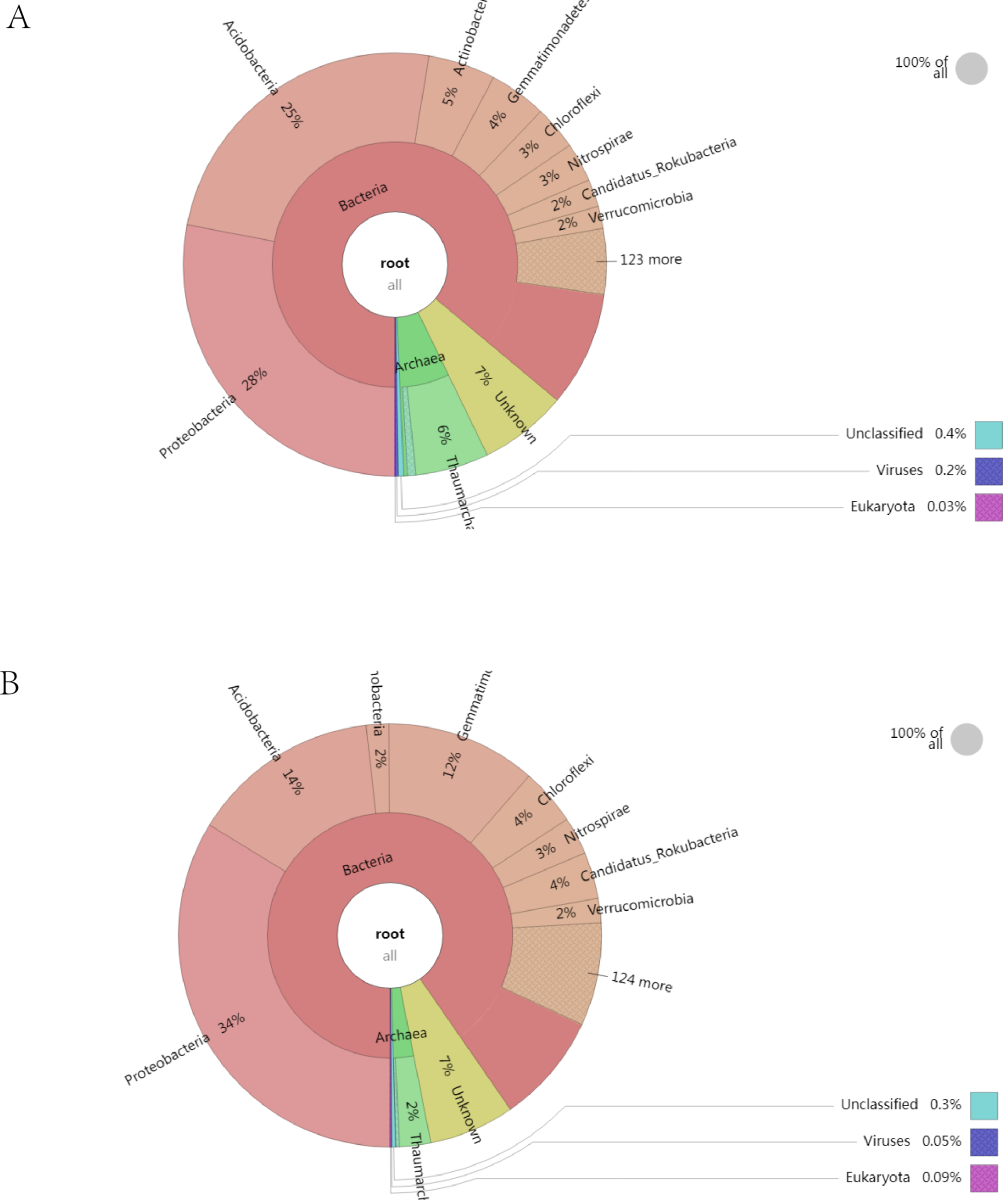
The microbial composition structure of rhizosphere soil. (A) Microbial composition structure in rhizosphere soil of diseased plants. (B) Microbial composition structure in rhizosphere soil of healthy plants.

Rhizosphere soils of diseased and healthy plants were analyzed by sequencing using Illumina, and a total of 25.95 G of clean data was obtained. Clean data were assembled and analyzed, and a total of 778,013 contigs were obtained with a total length of 501,277,738 bp. The gene prediction results based on scaftigs showed that a total of 867,164 genes were obtained, and the total length of genes in the gene catalog was 442.66 Mbp, with an average length of 509.56 bp. Overall, bacteria accounted for the vast majority in the rhizosphere soil of both healthy and diseased plants, with 90% (healthy plants) and 86% (diseased plants), respectively. Followed by archaea and viruses, eukaryota are very few. Krona analysis showed that Proteobacteria, Acidobacteria, Gemmatirhonadetes, Thaumarchaeota, Actinobacteria, Chloroflexi and Candidatus Rokubacteria were the dominant phyla in the rhizosphere soil. Among them, Proteobacteria and Gemmatirhonadetes had higher relative abundances in the rhizosphere soil of healthy plants than that of diseased plants. However, the abundance of Acidobacteria, Thaumarchaeota and Actinobacteria in the rhizosphere soil of diseased plants was higher than that of healthy plants (Fig. 1). A total of 139 species of virus were detected in the rhizosphere soil of diseased and healthy plants, including 107 species of virus in the rhizosphere soil of diseased plants and 70 species of virus in the rhizosphere soil of healthy plants (Table S1). A total of five bacteriophages including RSL1, DU_RP_I, DU_RP_II, RSK1 and RSP15 targeting *Ralstonia solanacearum* were detected, which was consistent with previous publications and links in the National Center for Biotechnology Information database (23–27). Among them, the relative abundance of bacteriophage DU_RP_I was higher in the rhizosphere soil of healthy plants, while the abundance of the other four bacteriophages was higher in the rhizosphere soil of diseased plants. However, further investigation is required to determine the specific factors underlying this observed variation. In addition, a total of 71 fungi were detected in the rhizosphere soil of diseased and healthy plants, and no common pathogenic fungi of tobacco root diseases were found.

### Differential genera analysis of rhizosphere soil of healthy and diseased plants

**Fig 2:**
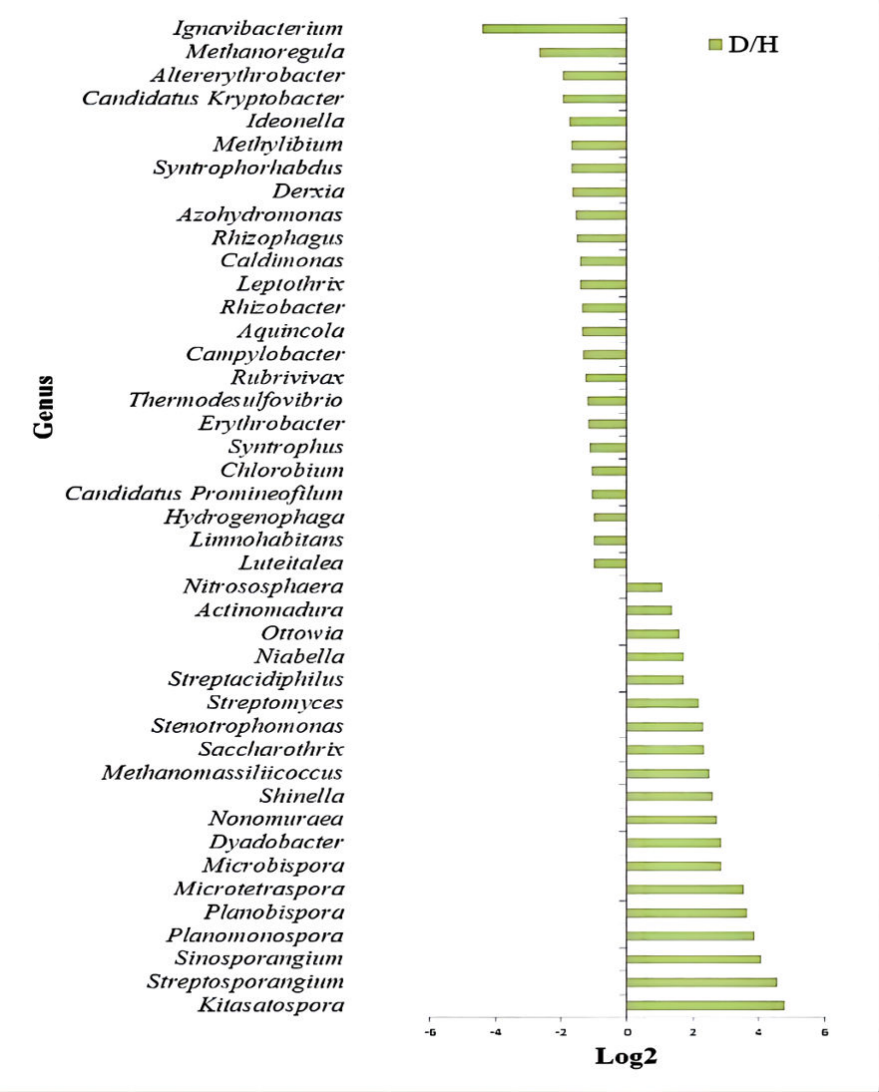
Comparison of rhizosphere soil microbial differences at genus level between diseased (D) and healthy (H) plants.

In order to better understand the differences in soil microbial composition, the study further explored the key driver microbiota. Here, we used the linear discriminant analysis (LDA) scores more than twofold as the screening criterion, and finally, 43 microbial genera that have a significant difference between the rhizosphere soil of diseased and healthy plants were screened. The results of linear discriminant analysis effect size (LEfSe) showed that compared with the healthy plant, the relative abundance of 19 genera in the rhizosphere soil of diseased plants were significantly increased, of which 12 belonged to Actinomycetes, including *Actinomadura*, *Streptacidiphilus*, *Streptomyces*, *Saccharothrix*, *Nonomuraea*, *Microbispora*, *Microtetraspora*, *Planobispora*, *Planomonospora*, *Sinosporangium*, *Streptosporangium* and *Kitasatospora* (Fig. 2). However, 24 bacterial genera were significantly enriched in the rhizosphere soil of healthy plants, most of which (16 genera) belonged to Proteobacteria, and the remaining 8 belonged to different phyla (Fig. 2).

### Functional analysis based on unigenes

**Fig 3:**
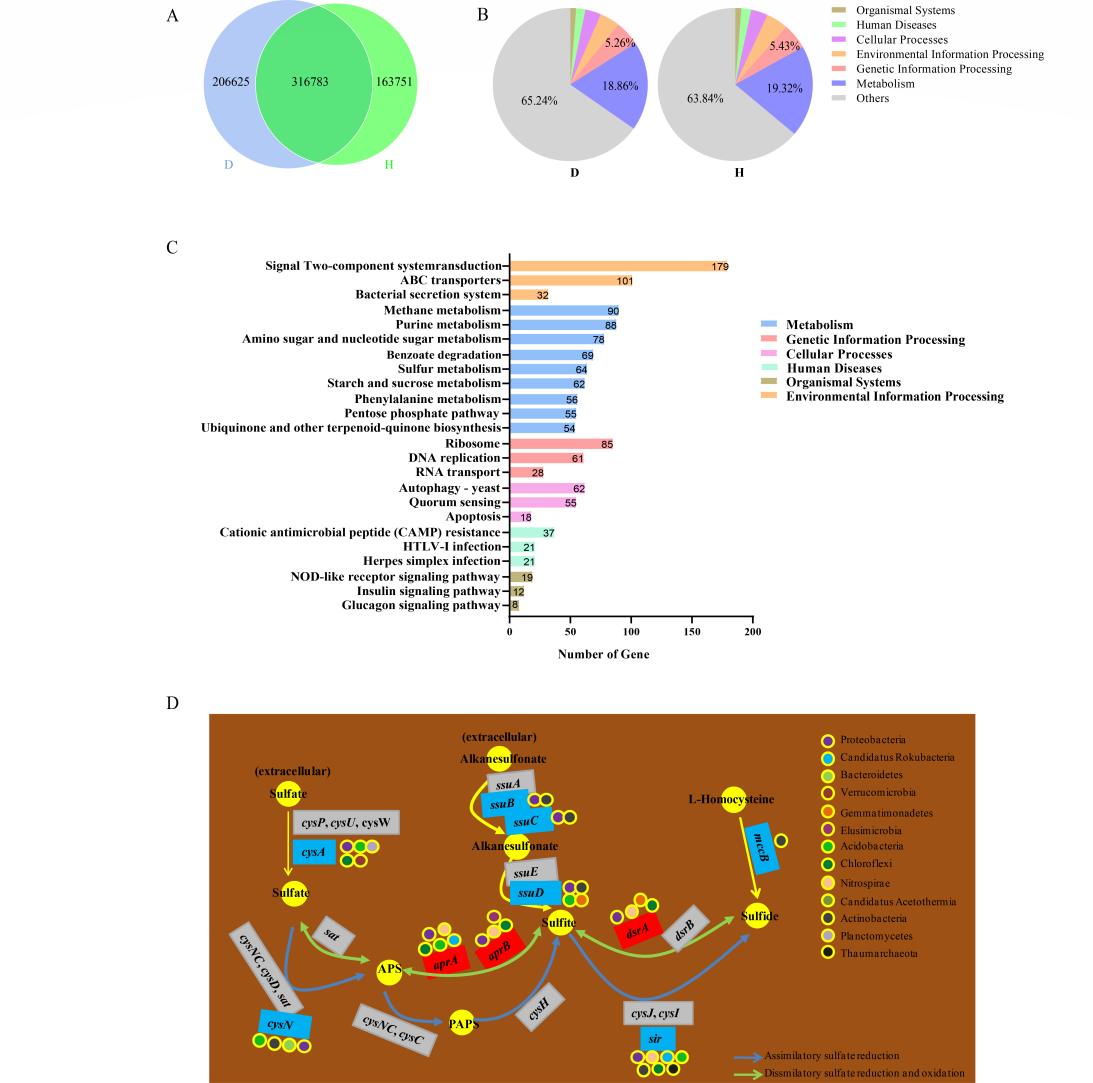
Metagenomic functional analysis of healthy and diseased soils. (**A)** Comparison of metagenomic ORF annotations between healthy and diseased soils. (B) Unigene classification of healthy and diseased soil. (C) KEGG enrichment of genes with differential abundance between healthy and diseased soil. (D) Schematic representation of the flow of sulfur in soil. The blue box represents the high abundance level of genes in diseased soil; the red box represents the high abundance level of genes in healthy soil; the gray box represents the insignificant difference in gene abundance between two soils; and the colored circle represents the phylum, where the gene is detected. APS, adenosine 5′-phosphosulfate.

After understanding the structure of soil microbial communities, we further focused on the functions of different soil microbiomes. Metagenomic sequencing results showed that a total of 286,685,251 bp was detected in the rhizosphere soil samples of the diseased plants, and 486,506 open reading frames (ORFs) were predicted. A total of 214,592,487 bp was detected in the rhizosphere soil samples of the diseased plants, and 380,658 ORFs were predicted. Among them, the number of ORFs shared in the rhizosphere soil of diseased and healthy plants was 316,783, while the unique ORFs of each soil were 206,625 and 163,751 respectively (Fig. 3A). A total of 412,863 unigenes were identified in two kinds of soil, which included 185,780 unigenes in the rhizosphere soil of diseased plants and 227,803 unigenes in the rhizosphere soil of healthy plants. These unigenes can be mapped to 5,198 KEGG orthology groups. KEGG function annotation of these unigenes shows that most of the functions of these unigenes are unknown. The most unigenes with known functions are mapped to the metabolism class (Fig. 3B). The differential unigenes in rhizosphere soils of healthy and diseased plants were mainly enriched in the KEGG pathways shown in Fig. 3C. We found that the differential unigenes in the two soils were significantly enriched in the sulfur metabolism pathway, and the microorganisms in the rhizosphere soil of the diseased plants were better at transferring extracellular sulfur-containing substances into the cells (Fig. 3D). For example, bacteria expressing the *cysA* gene can transfer sulfate from bacterial extracellular to intracellular, while bacteria expressing the *ssuB* and *ssuC* genes can transfer alkanesulfonate from bacterial extracellular to intracellular (Fig. 3D). In addition, the *cysN* and *sir* genes enriched in the rhizosphere of diseased plants are involved in assimilating sulfate reduction, while the *aprA*, *aprB*, and *dsrA* genes enriched in the rhizosphere of healthy plants are involved in dissimilated sulfate reduction (Fig. 3D). Of these, *aprA* is primarily sourced from the Proteobacteria, with minor contributions from Nitrospirae, Chloroflexi, Candidatus Rokubacteria and Acidobacteria; *aprB* and *dsrA* predominantly originate from the Proteobacteria and Nitrospirae; *CysN* mainly comes from Proteobacteria and Acidobacteria; *Sir* is primarily sourced from Acidobacteria and Thaumarchaeota, with minor contributions from Proteobacteria, Nitrospirae, Candidatus Rokubacteria and Actinobacteria. Therefore, we speculate that the rhizosphere soil of diseased plants was better at conducting soil sulfur cycling through assimilatory sulfate reduction, while the rhizosphere soil of healthy plants was better at conducting soil sulfur cycling through dissimilatory sulfate reduction and oxidation. Moreover, we analyzed the involvement of microorganisms in soil nitrogen cycling in two kinds of soils (Fig. S1). The results showed that four functional genes involved in nitrogen cycling were enriched in the rhizosphere of healthy plants, namely, *nifD*, *nirS*, *ncd2* and *hcp*. Among them, *nifD* gene is associated with microbial nitrogen fixation, *nirS* gene is associated with nitrite reduction, *ncd2* gene is associated with ammonification, and *hcp* gene is associated with hydroxylamine reduction (Fig. S1).

In addition, eggNOG and CAZY function annotations were performed on these unigenes. The eggNOG annotation results showed that most of the functions of unigenes detected by metagenome were unknown, and unigenes with known functions have a relatively high proportion in energy production and conversion, amino acid transport and metabolism, carbohydrate transport and metabolism, and replication, recombination and repair (Fig. S2). CAZY annotation results showed that the functions of unigenes were mainly concentrated in the glycoside hydrolases, glycosyl transferases, carbohydrate-binding modules and carbohydrate esterases categories, while auxiliary activities and polysaccharide lyases accounted for a relatively small proportion (Fig. S3).

### Comparison of rhizosphere soil antibiotic resistance genes between diseased and healthy plants

**Fig 4:**
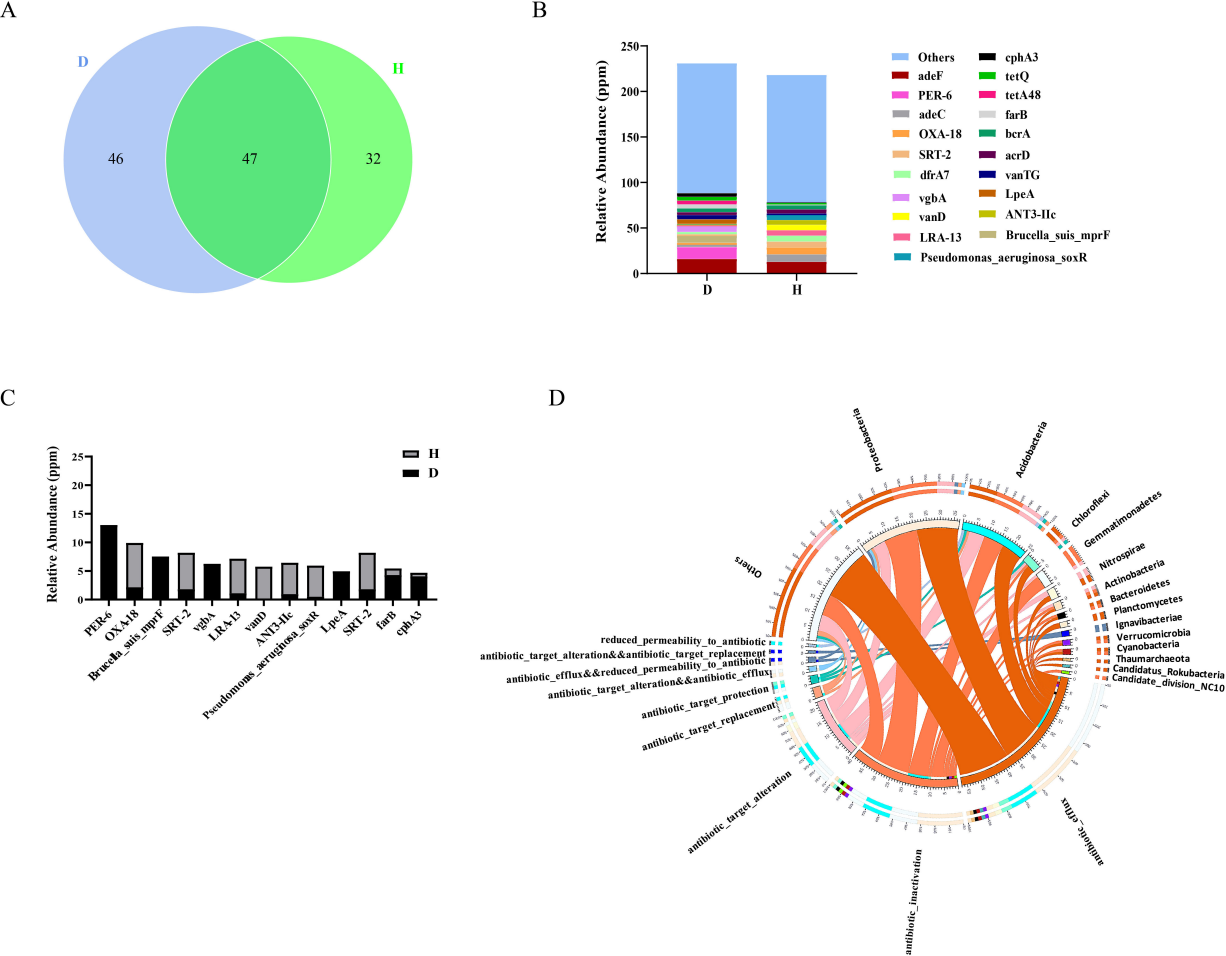
Differences in resistance genes between diseased (D) and healthy (H) soils. (A) Comparison of resistance gene annotation between healthy and diseased soils. (B) Classification of resistance genes in healthy and diseased soils. (C) Differences in the abundance of resistance genes between healthy and diseased soils (top 15). (D) Overview of the distribution proportion of resistance gene types in each sample (the right side shows the sample information).

The analysis results in Fig. 2 showed that more than half of the differential microorganisms enriched in the rhizosphere soil of the diseased plants belonged to Actinomycetes, and the production of antibiotics was one of the characteristics of Actinomycetes. This finding led us to hypothesize that other microorganisms coexisting with these Actinomycetes might possess antibiotic resistance. To test this hypothesis, we aligned the gene sets obtained by metagenomic sequencing in the Comprehensive Antibiotic Resistance Database (CARD). The results showed that 150 unigenes were mapped to the CARD database and associated with 125 antibiotic resistance ontologies (AROs). Among them, the number of AROs shared in the rhizosphere soil of diseased and healthy plants was 47, while the unique AROs of each soil were 46 and 32, respectively (Fig. 4A). Subsequently, we conducted an inter-group differential analysis on the abundance of resistance genes in two soil samples. The results showed that the overall abundance of resistance genes contained in the rhizosphere soil of diseased plants was relatively higher than that of healthy plants (Fig. 4B). The abundance of resistance genes shown in Fig. 4C was significantly different between the two kind of soils, including PER_6, OXA18, brucellaBrucella_suis_mprF, SRT-2 and nine other genes. Figure 4D illustrates the correspondence between the function of resistance genes and microorganisms. Therefore, compared with the rhizosphere soil of healthy plants, the rhizosphere soil of diseased plants may possess a stronger degradation potential for cephalosporins, peptide antibiotics, streptococcin antibiotics, macrolide antibiotics, antibacterial free fatty acids, tetracyclic antibiotics, etc.

### The correlation between soil properties and pathogen content

**Fig 5:**
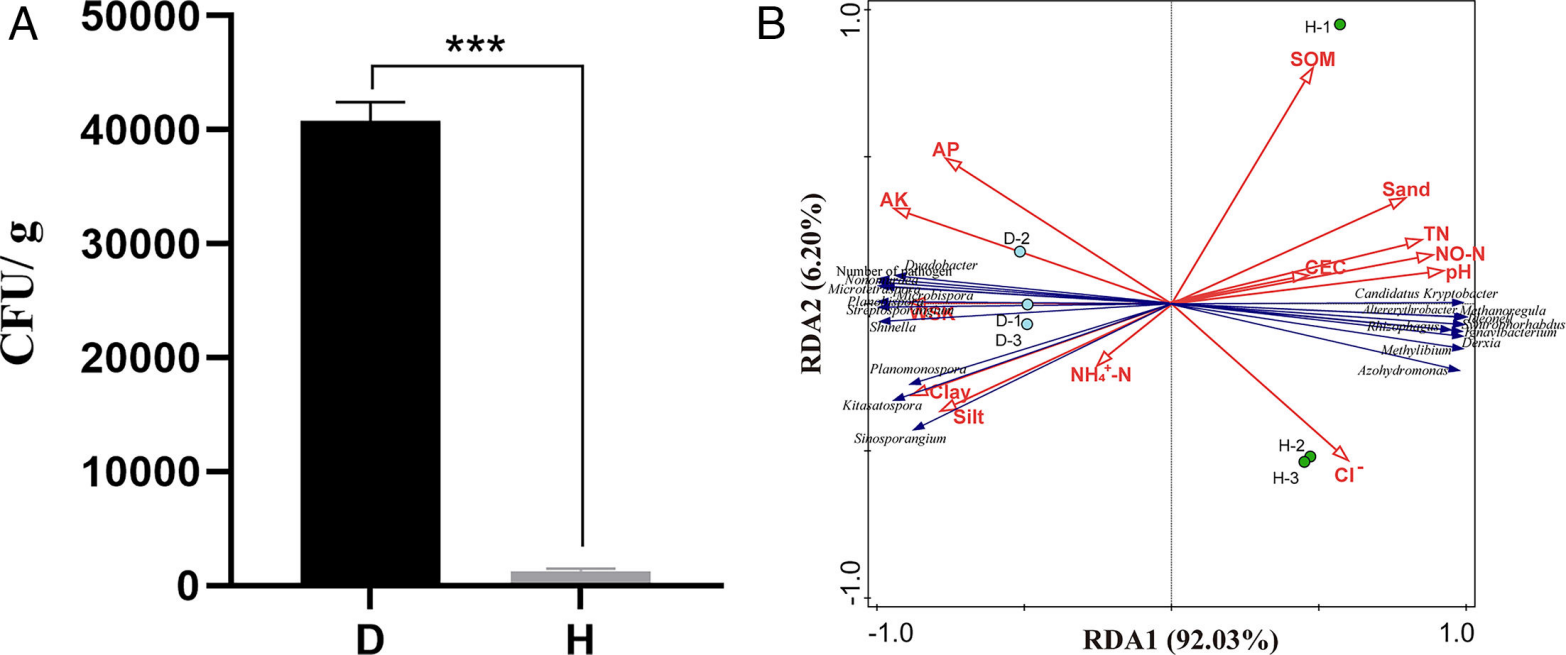
The correlation between soil properties and the amount of *R. solanacearum*. (A) Quantity of the pathogen in healthy and diseased soil. (B) Redundancy analysis (RDA) diagram of soil properties and the amount of *R. solanacearum*. All of the environmental variables (blue lines with arrows) shown were tested by partial Monte Carlo permutations at the *P* < 0.05 level and selected according to their marginal effects in descending order. Clay, particle size <0.5 mm; silt, particle size 0.005–0.05 mm; sand, particle size 0.05–1.0 mm. AK, exchangeable potassium; AP, available phosphorus; CEC, cation exchange capacity; Cl^−^, chloride ion; NH_4_^+^-N, nitrate nitrogen; NO-N, ammonium nitrogen; SOM, soil organic matter; TN, total nitrogen; WSK, water-soluble potassium.

To explore the impact of soil properties on the number of pathogens, we measured the amount of *R. solanacearum* present in the rhizosphere soil of healthy and diseased plants. The results showed that the number of *R. solanacearum* in the rhizosphere soil of diseased plants was about 30 times that of healthy plants rhizosphere soil (Fig. 5A). The results of redundancy analyses showed that the proportion of particles sizes of <0.5 mm in the rhizosphere soil was significantly positively correlated with the the number of *R. solanacearum* in the rhizosphere soil (Fig. 5B). Meanwhile, the amount of *R. solanacearum* in rhizosphere soil is negatively correlated with soil nitrate nitrogen, total nitrogen and pH, and positively correlated with exchangeable potassium and water-soluble potassium (Fig. 5B). In addition, the top 10 microorganisms enriched in the rhizosphere of healthy plants showed a significant positive correlation with soil pH and a negative correlation with water-soluble potassium (Fig. 5B). Based on the above results, we believe that soil properties are related to the enrichment of specific bacteria in the soil.

## DISCUSSION

In this study, we conducted an analysis of abundance and diversity of microorganisms in the rhizosphere soil of both diseased and healthy plants based on metagenomic sequencing results. Bacteria accounted for the vast majority in the two kinds of soil. The relative abundance of Proteobacteria and Gemmatirhonadetes in the rhizosphere soil of healthy plants was higher than that in the rhizosphere soil of diseased plants, and the relative abundance of Acidobacteria in the rhizosphere soil of healthy plants was lower than that in the rhizosphere soil of diseased plants. These findings align with prior studies conducted on the rhizosphere of both bacterial wilt-resistant and susceptible tomato plants (28). Furthermore, our results showed that a higher abundance of Actinomycetes in the rhizosphere soil of diseased plants compared to healthy plants, which is contrary to previous research on tomatoes. This may be because Actinobacteria contains a very large number of genera and species, each with vastly different functions. Therefore, we further analyzed the differences between the rhizosphere soil of diseased and healthy plants at the genus level, and a total of 43 genera with significant differences were screened. Among them, *Ignavibacterium* enriched in the rhizosphere soil of healthy plants has been shown to be enriched in soil with low incidence of pepper blight (29); *Altererythritobacter* and *Rhizobacter* enriched in the rhizosphere soil of healthy plants were linked to suppression of tomato *Fusarium* wilt disease (30, 31); *Ideonella* enriched in the rhizosphere soil of healthy plants reduced the virulence of *R. solanacearum* (32); and bacteria belonging to *Rhizophagus* have been shown to reduce the incidence of tomato bacterial wilt (33). Therefore, we speculate that some beneficial microorganisms related to disease resistance were enriched in the rhizosphere soil of healthy plants.

Compared with the rhizosphere soil of healthy plants, of 19 genera enriched in the rhizosphere soil of diseased plants, 13 belonged to Actinomycetes, and most of these Actinomycetes can produce antibiotics (34–37). The results based on CARD database showed that the overall abundance of resistance genes contained in the rhizosphere soil of diseased plants was relatively higher than that of healthy plants (Fig. 4B). In other words, the rhizosphere soil of diseased plants contains more microorganisms that produce antibiotics and also contains more microorganisms that can resist antibiotics.

Soil microorganisms are closely related to nutrient cycling during plant growth, including soil carbon sequestration, nitrogen conversion and sulfur cycling. The results of this study showed that the differential unigenes in the rhizosphere soil of diseased and healthy plants were significantly enriched in the sulfur metabolism pathway (Fig. 3C). The rhizosphere soil microorganisms of diseased plants were better at conducting sulfur cycling through assimilatory sulfate reduction, while the rhizosphere soil microorganisms of healthy plants were better at conducting sulfur cycling through dissimilatory sulfate reduction and oxidation (Fig. 3D). Sulfur participates in many key physiological and biochemical processes in plants, such as aerobic respiration, protein synthesis and biological nitrogen fixation, which is an important factor in determining the productivity and quality of agricultural products (38). S^+6^ applied as fertilizer or obtained through the processes described above is the form of sulfur that plants assimilate through their roots (38). The oxidation from S^0^ to S^+6^ is carried out by most soil microorganisms, highlighting *Thiobacillus*, *Beggiatoa*, *Desulfomicrobium* and *Desulfovibrio*, as well as other heterotrophic aerobics S^−^ oxidizing bacteria such as *Bacillus*, *Pseudomonas* and *Arthrobacter* (39, 40). Our study found that multiple genera involved in sulfur transformation are enriched in the rhizosphere soil of healthy plants, including *Thermodesulfovibrio*, *Syntrophorhabdus*, *Syntrophus*, *Chlorobium*, *Hydrogenophaga* and *Limnohabitans* (41–45). Among them, *Chlorobium*, *Hydrogenophaga* and *Limnohabitans* were involved in generate sulfate from elemental S (43, 45). Sulfur has also been reported to be associated with plant tolerance to biotic and abiotic stresses (38). Therefore, we speculate that the differences in microorganisms in the rhizosphere soil of healthy and diseased plants lead to differences in the form of sulfur elements, which indirectly affect the plant’s tolerance to pathogens.

The analysis of unigene function in the two types of soil also showed that the number of functional unigenes involved in the nitrogen cycling (especially the nitrogen fixation pathway) in the rhizosphere soil of healthy plants was significantly higher than that of diseased plants rhizosphere soil (Fig. S1). Nitrogen plays an important role in plant growth, and the available nitrogen content in the soil is one of the limiting factors for the productivity of terrestrial ecosystems (46). The process of soil nitrogen cycling is mainly driven by microorganisms, and our research showed that the microorganisms enriched in rhizosphere soil of healthy plants contained multiple microorganisms involved in soil nitrogen cycling, such as *Ignavibacterium*, *Ideonella*, *Derxia* and *Azohydromonas*. Among them, *Derxia* and *Azohydromonas* are involved in soil nitrogen fixation (47, 48). Previous studies have shown that the load of microbial nitrogen metabolism genes in the rhizosphere soil significantly varied after the infection, resulting in a stronger denitrification process in the diseased soil, which is consistent with our findings (10). In addition, Ding and colleagues studied the interaction between tomato and pathogens (*R. solanacearum*) under four nitrogen regimes, and their findings indicated that nitrogen forms and metabolism affected the plant defense against pathogens in tomato (49). In this study, the contents of ammonia, nitrate and total nitrogen in the rhizosphere soil of diseased and healthy plants were measured. The results showed that the contents of nitrate nitrogen and total nitrogen in the rhizosphere soil of healthy plants were significantly higher than those in the rhizosphere soil of diseased plants and were negatively correlated with the number of *R. solanacearum* in the soil (Fig. 5). Therefore, we speculate that the nitrogen forms and abundance of nitrogen metabolism-related genes in the soil influence the defense of plants against *R. solanacearum*.

A previous study has demonstrated differences in soil chemical properties between healthy and bacterial wilt-infected soils, with the available potassium, available phosphorus and pH levels in the latter being significantly lower than those in healthy soils (50). In a separate investigation, Shi and his colleagues found that the pH of healthy soil was significantly higher than soil impacted by bacterial wilt disease across multiple tobacco planting areas, but the changes in available potassium content was not completely consistent in the studied regions (51). Our study’s findings revealed that the available phosphorus and pH levels in the rhizosphere soil of diseased plants were significantly lower than those in healthy plants, while the exchangeable potassium was higher in the rhizosphere soil of diseased plants compared to healthy plants (Table 1). The research of Wang and colleagues found that there were significant differences in the content of ammonia nitrogen, hydrolyzed nitrogen and organic matter between healthy and bacterial wilt-infected soils (5). Our study suggested that there were no significant differences in ammonium nitrogen, cation exchange capacity, chloride ions, organic matter and hydrolyzed nitrogen between the rhizosphere soil of diseased and healthy plants (Table 1). In addition, we analyzed the difference in rhizosphere soil particle size between diseased plants and healthy plants. The result showed that there is a significant difference in the proportion of particle sizes 0.005–0.05 mm and 0.05–1.0 mm in the rhizosphere soil of diseased and healthy plants (Table 2). Redundancy analysis showed that there was a significant correlation between soil particle size and the number of *R. solanacearum* (Fig. 5B). In summary, we believe that the soil physicochemical properties, the forms of sulfur and nitrogen elements, and the composition of host rhizosphere microbial communities interact to jointly affect the occurrence of tobacco bacterial wilt. Our findings here will provide more mechanistic explanations for the occurrence of tobacco bacterial wilt and contribute to identification of more effective measures for the prevention and control of bacterial wilt.

## MATERIALS AND METHODS

### Collection of rhizosphere soil samples

The *Nicotiana tabacum* cultivar Yunyan 87 planted in the field situated at Lichuan County, Fuzhou City, Jiangxi Province, China, was used as for collection of samples, and the roots of 15 bacterial wilt-diseased plants and 15 healthy plants from fields were collected. Every five roots were pooled into one sample for subsequent experiments. Healthy plants were collected from fields that had been disease-free for nearly 5 years. The bulk soil and the impurities attached to the tobacco root surface were removed, and then the roots were shortened to about 10 cm. The shortened roots were placed into a 500-mL centrifuge bottle, treated with ultrasonic waves at 70 Hz for 30 min, and shaken at 28°C for 30 min. The root system was removed and the soil remaining in the centrifuge bottle was stored, which was the tobacco rhizosphere soil sample.

### Measurement of soil physicochemical properties and *R. solanacearum* quantity

Soil particle size was determined by laser particle size analyzer (HELOS/OASISType, SYMPATEC Gmbh, Germany). Soil total organic matter levels were determined using the high-temperature heating potassium dichromate oxidation volumetric method (52). The determination of soil total nitrogen was carried out according to the semi-micro Kelvin method. Soil nitrate nitrogen and ammonium nitrogen were detected by the flow injection method. The hydrolyzable nitrogen was measured by the alkaline diffusion method and extracted with 1.5-mol/L NaOH. Available phosphorus was extracted with 0.5-M NaHCO_3_ (pH 8.5) according to Olsen et al. (53). Exchangeable potassium was extracted by shaking the soil in 0.1-M BaCl_2_, and then the extracted elements were analyzed with flame atomic absorption spectrometry (Varian AA280FS). Water-soluble potassium was extracted from air-dried soil by shaking in 100 mL of deionized water, and the potassium content was subsequently determined using a flame photometer (AP-1200, Precision Instrument Co., Ltd., Shanghai, China). Water dissolving chloride ions was determined by potassium chromate colorimetric method. The cation exchange capacity was extracted with 1.0-M ammonium acetate at pH 7.0 (54). The pH value of the soil was measured by the potentiometer method, and the water:soil ratio was 4:1.

Quantitative polymerase chain reaction (qPCR) was used to detect the abundance of *R. solanacearum* in soil. *R. solanacearum* mode strain GMI1000 was activated and cultured at 28°C. Culture broths containing different concentrations of *R. solanacearum* were prepared, and the total DNA of each concentration was extracted. A standard curve based on the concentration of *R. solanacearum* was established by qPCR using ddH_2_O as the blank control, and qPCR was performed with specific primers 759F: GTCGCCGTCAACTCACTTTCC and 760R: GTCGCCGTCAGCAATGCGGAATCG. Soil genomic DNA was extracted, and qPCR was performed with the same procedure. The obtained cycle threshold values were brought into the standard curve to calculate the abundance of pathogenic bacteria in the soil.

### Extraction and sequencing of metagenomic DNA

Metagenomic DNA from the rhizosphere soil of diseased and non-diseased plants was extracted as described in Stevenson et al. (55). DNA (30–50 ng) was sonicated to a 100- to 800-bp size range using the Covaris ME220 (Covaris, USA). DNA fragments were subsequently end repaired and 3′-adenylated before Illumina adapters were added by using the NEBNext Sample Reagent Set (New England Biolabs). Ligation products were purified by Agencourt AMPure XP Nucleic Acid Purification Kit (Beckman Coulter, Germany). High-quality libraries were constructed using the Next Ultra IIDNA Library Preparation Kit (NEB, UK), and DNA fragments were PCR-amplified using Next Q5 Hot Start HiFi PCR PreMix (NEB, UK). After library profile analysis using an Agilent 2100 Bioanalyzer (Agilent Technologies, USA) and qPCR quantification (MxPro, Agilent Technologies), each library was sequenced using 101-base length read chemistry in a paired-end flow cell on Illumina sequencing machines (Illumina, USA).

### Metagenomic sequencing data processing

#### Metagenome sequence quality control and assembly

After the original data were obtained by sequencing, low-quality reads were filtered using Usearch software (http://www.drive5.com/usearch/). Paired-end reads were spliced into tags, and low-quality tags were filtered. The data obtained were called clean tags. Megahit (https://github.com/voutcn/megahit) was used for metagenome assembly from the clean data after quality controls, and the unused reads of each sample were mixed together for the discovery of low-abundance species information in the sample (56).

#### Gene predictions

Gene coding sequences (minimum length 100 nt) were predicted on the assembled scaftigs [option gene_prediction, MetaGeneMark (version 2.8)] (57). MetaGeneMark was used for gene prediction, and the genes generated by each sample and mixed assembly prediction were put together to remove redundancy and construct a gene catalog. The abundance information of the gene catalog in each sample can be obtained by combining the clean data of each sample. Using BWA software, the clean data were aligned to the non-redundant gene catalog (58), and the abundance information of each gene in each sample was calculated from the number of reads and gene length on the alignment (59). The calculation formula was as follows:


$$\text{RPKM =}\frac{\text{Number of reads mapped to gene}\times {\text{10}}^{\text{3}}\times {\text{10}}^{\text{6}}}{\text{Total number of mapped reads}\times \text{gene length in bp}}.$$

#### Functional annotation of gene catalog

The gene catalog was functionally annotated to orthologous groups in the eggNOG (version 3) and KEGG databases (version 62) using SmashCommunity (version 1.6) (60). Gene abundance profiles were generated by mapping high-quality reads from each sample to the gene catalog. The abundance distribution, species attribution and resistance mechanism of these resistance genes can be obtained by annotating the gene catalog and the CARD.

#### Species annotation

The species annotation information of each gene (Unigene) was obtained by comparing with the MicroNR library. The alignment sequence with the highest similarity was taken as the species annotation information of the sequence, and the sum of the abundances of ORFs that could be annotated to the same species was calculated and normalized to 100% as the relative abundance of the species. Subsequently, the abundance of species in each sample was calculated at the taxonomic levels of domain, kingdom, phylum, class, order, family, genus and species, so as to construct the abundance profile at the taxonomic level.

### Metagenomic sequencing data analysis

According to the analysis process, species composition analysis, indicator species analysis, community function prediction and gene functional profiling were performed. Redundancy analysis, correlation analysis and linear correlation analysis, combined with other factors (such as environmental factors), were carried out to determine the relationships between microorganisms and the environment. LDA score and evolutionary branching diagrams were analyzed using the “LEfSe”package in the Python 2 environment. Redundancy analysis was performed using Canoco (version 5) software. The species abundance stack map and gene distribution pie chart were plotted using GraphPad Prism (version 8). The difference in the amount of the pathogen in the rhizosphere soil between diseased and healthy plants was analyzed using Student’s *t*-test (two tailed) with IBM SPSS (version 20).

## Data Availability

The data are available at GenBank under the BioProject ID: PRJNA1142923 and PRJNA1142924.

## SUPPLEMENTAL MATERIAL

Supplemental figures spectrum.00559-24-s0001.pdf Fig. S1 to S3.

Table S1 spectrum.00559-24-s0002.xlsx Annotated results of viruses in rhizosphere soil of healthy and diseased plants.

ASM does not own the copyrights to Supplemental Material that may be linked to, or accessed through, an article. The authors have granted ASM a non-exclusive, world-wide license to publish the Supplemental Material files. Please contact the corresponding author directly for reuse.

## References

[1] Hu Y, Zhao W, Li X, Feng J, Li C, Yang X, Guo Q, Wang L, Chen S, Li Y, Yang Y. 2021. Integrated biocontrol of tobacco bacterial wilt by antagonistic bacteria and marigold. Sci Rep 11:16360. doi:10.1038/s41598-021-95741-w 34381095 PMC8357815

[2] Jiang G, Wei Z, Xu J, Chen H, Zhang Y, She X, Macho AP, Ding W, Liao B. 2017. Bacterial wilt in china: history, current status, and future perspectives. Front Plant Sci 8:1549. doi:10.3389/fpls.2017.01549 28955350 PMC5601990

[3] He MX, Shen L, Qiu HL, Zhang RK, Chen BW, Chen SM. 2019. Occurrence and control techniques of tobacco bacterial wilt. Mod Agri Sci Tech (China)

[4] Zu QX, Nie ZY, Lin S, Ran C, Zhang YF, Yang C, Liu J. 2022. Advances in control of tobacco bacterial wilt. Plant Health Med (China) 1:90–96.

[5] Wang R, Zhang H, Sun L, Qi G, Chen S, Zhao X. 2017. Microbial community composition is related to soil biological and chemical properties and bacterial wilt outbreak. Sci Rep 7:343. doi:10.1038/s41598-017-00472-6 28336973 PMC5428506

[6] Ahmed W, Dai Z, Zhang J, Shakeel Q, Kamaruzzaman M, Nosheen S, Mohany M, Ahmed A, Cai S, Wang Y, Gao Y, Ahmad M, Munir S, Wang X. 2024. Ralstonia solanacearum differentially modulates soil physicochemical properties and rhizospheric bacteriome of resistant and susceptible tobacco cultivars. Microbiol Res 281:127604. doi:10.1016/j.micres.2024.127604 38280370

[7] Wei Z, Hu J, Gu Y, Yin SX, Xu YC, Jousset A, Shen QR, Friman VP. 2018. Ralstonia solanacearum pathogen disrupts bacterial rhizosphere microbiome during an invasion. Soil Biol Biochem 118:8–17. doi:10.1016/j.soilbio.2017.11.012

[8] Wu QY, Ma R, Wang X, Ma YN, Wang ZS, Wei HL, Zhang XX. 2024. Effects of the invasion of Ralstonia solanacearum on soil microbial community structure in Wuhan, China. mSphere 9:e0066523. doi:10.1128/msphere.00665-23 38231250 PMC10900898

[9] Su L, Zhang L, Nie D, Kuramae EE, Shen B, Shen Q. 2020. Bacterial tomato pathogen Ralstonia solanacearum invasion modulates rhizosphere compounds and facilitates the cascade effect of fungal pathogen Fusarium solani. Microorganisms 8:806. doi:10.3390/microorganisms8060806 32471167 PMC7356623

[10] Wang Z, Zhang Y, Bo G, Zhang Y, Chen Y, Shen M, Zhang P, Li G, Zhou J, Li Z, Yang J. 2022. Ralstonia solanacearum infection disturbed the microbiome structure throughout the whole tobacco crop niche as well as the nitrogen metabolism in soil. Front Bioeng Biotechnol 10:903555. doi:10.3389/fbioe.2022.903555 35800334 PMC9253565

[11] Zhang T, Chen HYH, Ruan H. 2018. Global negative effects of nitrogen deposition on soil microbes. ISME J 12:1817–1825. doi:10.1038/s41396-018-0096-y 29588494 PMC6018792

[12] Badri DV, Vivanco JM. 2009. Regulation and function of root exudates. Plant Cell Environ 32:666–681. doi:10.1111/j.1365-3040.2008.01926.x 19143988

[13] Yang C, Zhang C, Dittman JD, Whitham SA. 2009. Differential requirement of ribosomal protein S6 by plant RNA viruses with different translation initiation strategies. Virol (Auckl) 390:163–173. doi:10.1016/j.virol.2009.05.018 19524993

[14] Trivedi P, Leach JE, Tringe SG, Sa T, Singh BK. 2020. Plant-microbiome interactions: from community assembly to plant health. Nat Rev Microbiol 18:607–621. doi:10.1038/s41579-020-0412-1 32788714

[15] Zhang Y, Hu A, Zhou J, Zhang W, Li P. 2020. Comparison of bacterial communities in soil samples with and without tomato bacterial wilt caused by Ralstonia solanacearum species complex. BMC Microbiol 20:89. doi:10.1186/s12866-020-01774-y 32290811 PMC7155298

[16] Peralta AL, Sun Y, McDaniel MD, Lennon JT. 2018. Crop rotational diversity increases disease suppressive capacity of soil microbiomes. Ecosphere 9:e02235. doi:10.1002/ecs2.2235

[17] Koga K, Hara H, Tanaka H. 1997. Suppressive soils to bacterial wilt of tobacco in Japan and population dynamics of Pseudomonas solanacearum in these soils. Jpn J Phytopathol 63:304–308. doi:10.3186/jjphytopath.63.304

[18] Zhai X, Zhang L, Wu R, Wang M, Liu Y, Lian J, Munir MAM, Chen D, Liu L, Yang X. 2022. Molecular composition of soil organic matter (SOM) regulate qualities of tobacco leaves. Sci Rep 12:15317. doi:10.1038/s41598-022-19428-6 36097148 PMC9468172

[19] Högberg MN, Högberg P, Myrold DD. 2007. Is microbial community composition in boreal forest soils determined by pH, C-to-N ratio, the trees, or all three? Oecologia 150:590–601. doi:10.1007/s00442-006-0562-5 17033802

[20] Tang L, Xia Y, Fan C, Kou J, Wu F, Li W, Pan K. 2020. Control of Fusarium wilt by wheat straw is associated with microbial network changes in watermelon rhizosphere. Sci Rep 10:12736. doi:10.1038/s41598-020-69623-6 32728175 PMC7391731

[21] Borrero C, Trillas MI, Ordovás J, Tello JC, Avilés M. 2004. Predictive factors for the suppression of Fusarium wilt of tomato in plant growth media. Phytopathology 94:1094–1101. doi:10.1094/PHYTO.2004.94.10.1094 18943798

[22] Marschner P, Fu Q, Rengel Z. 2003. Manganese availability and microbial populations in the rhizosphere of wheat genotypes differing in tolerance to Mn deficiency. Z Pflanzenernähr Bodenk 166:712–718. doi:10.1002/jpln.200320333

[23] Fujiwara A, Fujisawa M, Hamasaki R, Kawasaki T, Fujie M, Yamada T. 2011. Biocontrol of Ralstonia solanacearum by treatment with lytic bacteriophages. Appl Environ Microbiol 77:4155–4162. doi:10.1128/AEM.02847-10 21498752 PMC3131639

[24] Park TH. 2018. Complete genome sequence of DU_RP_II, a novel Ralstonia solanacearum phage of the family Podoviridae. Arch Virol 163:269–271. doi:10.1007/s00705-017-3577-9 28965163

[25] Trotereau A, Boyer C, Bornard I, Pécheur MJB, Schouler C, Torres-Barceló C. 2021. High genomic diversity of novel phages infecting the plant pathogen Ralstonia solanacearum, isolated in Mauritius and Reunion islands. Sci Rep 11:5382. doi:10.1038/s41598-021-84305-7 33686106 PMC7940629

[26] Park TH. 2020. Complete genome sequence of bacteriophage (DU_RP_I) infecting Ralstonia solanacearum. GenBank, 2020. Available from: https://www.ncbi.nlm.nih.gov/nuccore/MF979559.1/

[27] Kawasaki T, Fujie M, Chatchawankanphanich O, Ogata H, Yamada T. 2016. Genome analysis of a phage RSP15 infecting the phytopathogen Ralstonia solanacearum. GenBank, 2016. Available from: https://www.ncbi.nlm.nih.gov/nuccore/LC121084.1/

[28] Kwak MJ, Kong HG, Choi K, Kwon SK, Song JY, Lee J, Lee PA, Choi SY, Seo M, Lee HJ, Jung EJ, Park H, Roy N, Kim H, Lee MM, Rubin EM, Lee SW, Kim JF. 2018. Rhizosphere microbiome structure alters to enable wilt resistance in tomato. Nat Biotechnol 36: 1100–1109. doi:10.1038/nbt.4232 30295674

[29] Li H, Cai X, Gong J, Xu T, Ding G, Li J. 2019. Long-term organic farming manipulated rhizospheric microbiome and Bacillus antagonism against pepper blight (Phytophthora capsici). Front Microbiol 10:342. doi:10.3389/fmicb.2019.00342 30873141 PMC6401385

[30] Tang T, Sun X, Liu Q, Dong Y, Zha M. 2022. Treatment with organic manure inoculated with a biocontrol agent induces soil bacterial communities to inhibit tomato Fusarium wilt disease. Front Microbiol 13:1006878. doi:10.3389/fmicb.2022.1006878 36687620 PMC9849813

[31] Siegel-Hertz K, Edel-Hermann V, Chapelle E, Terrat S, Raaijmakers JM, Steinberg C. 2018. Comparative microbiome analysis of a fusarium wilt suppressive soil and a Fusarium wilt conducive soil from the Châteaurenard region. Front Microbiol 9:568. doi:10.3389/fmicb.2018.00568 29670584 PMC5893819

[32] Shinohara M, Nakajima N, Uehara Y. 2007. Purification and characterization of a novel esterase (beta-hydroxypalmitate methyl ester hydrolase) and prevention of the expression of virulence by Ralstonia solanacearum*.* J Appl Microbiol 103:152–162. doi:10.1111/j.1365-2672.2006.03222.x 17584461

[33] Chave M, Crozilhac P, Deberdt P, Plouznikoff K, Declerck S. 2017. Rhizophagus irregularis MUCL 41833 transitorily reduces tomato bacterial wilt incidence caused by Ralstonia solanacearum under in vitro conditions. Mycorrhiza 27:719–723. doi:10.1007/s00572-017-0783-y 28585092

[34] Lazzarini A, Cavaletti L, Toppo G, Marinelli F. 2001. Rare genera of actinomycetes as potential producers of new antibiotics. Antonie Van Leeuwenhoek 79:399–405. doi:10.1023/A:1010287600557 11816986

[35] Pozzi R, Coles M, Linke D, Kulik A, Nega M, Wohlleben W, Stegmann E. 2016. Distinct mechanisms contribute to immunity in the lantibiotic NAI-107 producer strain Microbispora ATCC PTA-5024. Environ Microbiol 18:118–132. doi:10.1111/1462-2920.12892 25923468

[36] Del Carratore F, Iorio M, Pérez-Bonilla M, Schmidt K, Pérez-Redondo R, Sosio M, Macdonald SJ, Gyulev IS, Tsigkinopoulou A, Thomas GH, Genilloud O, Rodríguez-García A, Donadio S, Breitling R, Takano E. 2021. Multi-omics study of Planobispora rosea, producer of the thiopeptide antibiotic GE2270A. mSystems 6:e0034121. doi:10.1128/mSystems.00341-21 34156292 PMC8269224

[37] Inahashi Y, Iwatsuki M, Ishiyama A, Namatame M, Nishihara-Tsukashima A, Matsumoto A, Hirose T, Sunazuka T, Yamada H, Otoguro K, Takahashi Y, Omura S, Shiomi K. 2011. Spoxazomicins A-C, novel antitrypanosomal alkaloids produced by an endophytic actinomycete, Streptosporangium oxazolinicum K07-0460(T). J Antibiot (Tokyo) 64:303–307. doi:10.1038/ja.2011.16 21386848

[38] Fuentes-Lara LO, Medrano-Macías J, Pérez-Labrada F, Rivas-Martínez EN, García-Enciso EL, González-Morales S, Juárez-Maldonado A, Rincón-Sánchez F, Benavides-Mendoza A. 2019. From elemental sulfur to hydrogen sulfide in agricultural soils and plants. Molecules 24:2282. doi:10.3390/molecules24122282 31248198 PMC6630323

[39] Wilhelm Scherer H. 2009. Sulfur in soils. Z Pflanzenernähr Bodenk 172:326–335. doi:10.1002/jpln.200900037

[40] Huxtable RJ. 1986. Biochemistry of sulfur. Plenum Publishing Corp, New York, USA.

[41] Umezawa K, Kojima H, Kato Y, Fukui M. 2020. Disproportionation of inorganic sulfur compounds by a novel autotrophic bacterium belonging to nitrospirota. Syst Appl Microbiol 43:126110. doi:10.1016/j.syapm.2020.126110 32847785

[42] Jantharadej K, Mhuantong W, Limpiyakorn T, Mongkolsuk S, Sirikanchana K, Suwannasilp BB. 2020. Identification of sulfate-reducing and methanogenic microbial taxa in anaerobic bioreactors from industrial wastewater treatment plants using next-generation sequencing and gene clone library analyses. J Environ Sci Health A Tox Hazard Subst Environ Eng 55:1283–1293. doi:10.1080/10934529.2020.1789409 32657213

[43] Dinh HTT, Kambara H, Matsushita S, Aoi Y, Kindaichi T, Ozaki N, Ohashi A. 2022. Biological methane production coupled with sulfur oxidation in a microbial electrosynthesis system without organic substrates. J Environ Sci (China) 116:68–78. doi:10.1016/j.jes.2021.07.027 35219426

[44] Burn R, Misson L, Meury M, Seebeck FP. 2017. Anaerobic origin of ergothioneine. Angew Chem Int Ed Engl 56:12508–12511. doi:10.1002/anie.201705932 28786519

[45] Luo J, Tan X, Liu K, Lin W. 2018. Survey of sulfur-oxidizing bacterial community in the Pearl River water using soxB, sqr, and dsrA as molecular biomarkers. 3 Biotech 8:73. doi:10.1007/s13205-017-1077-y PMC5766448 29354384

[46] Fowler D, Coyle M, Skiba U, Sutton MA, Cape JN, Reis S, Sheppard LJ, Jenkins A, Grizzetti B, Galloway JN, Vitousek P, Leach A, Bouwman AF, Butterbach BK, Dentener F, Stevenson D, Amann M, Voss M. 2013. The global nitrogen cycle in the 21th century. Phil Trans R Soc B 368:20130165. doi:10.1098/rstb.2013.0164 23713126 PMC3682748

[47] Xie CH, Yokota A. 2004. Phylogenetic analyses of the nitrogen-fixing genus Derxia. J Gen Appl Microbiol 50:129–135. doi:10.2323/jgam.50.129 15486821

[48] Dahal RH, Chaudhary DK, Kim DU, Kim J. 2021. Azohydromonas caseinilytica sp. nov., a nitrogen-fixing bacterium isolated from forest soil by using optimized culture method. Front Microbiol 12:647132. doi:10.3389/fmicb.2021.647132 34093463 PMC8175650

[49] Ding S, Shao X, Li J, Ahammed GJ, Yao Y, Ding J, Hu Z, Yu J, Shi K. 2021. Nitrogen forms and metabolism affect plant defence to foliar and root pathogens in tomato. Plant Cell Environ 44:1596–1610. doi:10.1111/pce.14019 33547690

[50] Qi G, Ma G, Chen S, Lin C, Zhao X. 2019. Microbial network and soil properties are changed in bacterial wilt-susceptible soil. Appl Environ Microbiol 85:e00162-19. doi:10.1128/AEM.00162-19 31003986 PMC6581179

[51] Shi H, Xu P, Wu S, Yu W, Cheng Y, Chen Z, Yang X, Yu X, Li B, Ding A, Wang W, Sun Y. 2022. Analysis of rhizosphere bacterial communities of tobacco resistant and non-resistant to bacterial wilt in different regions. Sci Rep 12:18309. doi:10.1038/s41598-022-20293-6 36316337 PMC9622857

[52] Li CQ, Ren YJ, Zhao JH, Guo B, Liu LP. 2017. Potassium dichromate oxidation methods’ review and application for determination of soil organic matter. MEng 04:251–259. doi:10.12677/MEng.2017.44036

[53] Olsen SR, Watanabe FS, Dean LA, Cole CV. 1954. Estimation of available phosphorus in soils by extraction with sodium bicarbonate (USDA Circular 939). Government Printing Office, Washington, DC.

[54] Pansu M, Gautheyrou J. 2006. Handbook of soil analysis-mineralogical, organic and inorganic methods. Springer, Berlin Heidelberg, Berlin, Germany.

[55] Stevenson LJ, Ackerley DF, Owen JG. 2022. Preparation of soil metagenome libraries and screening for gene-specific amplicons. Methods Mol Biol 2397:3–17. doi:10.1007/978-1-0716-1826-4_1 34813056

[56] Li D, Liu CM, Luo R, Sadakane K, Lam TW. 2015. MEGAHIT: an ultra-fast single-node solution for large and complex metagenomics assembly via succinct de Bruijn graph. Bioinformatics 31:1674–1676. doi:10.1093/bioinformatics/btv033 25609793

[57] Zhu W, Lomsadze A, Borodovsky M. 2010. Ab initio gene identification in metagenomic sequences. Nucleic Acids Res 38:e132. doi:10.1093/nar/gkq275 20403810 PMC2896542

[58] Li H, Durbin R. 2009. Fast and accurate short read alignment with burrows–wheeler transform. Bioinformatics 25:1754–1760. doi:10.1093/bioinformatics/btp324 19451168 PMC2705234

[59] Lee J, Lee HT, Hong W, Jang E, Kim J. 2015. FCMM: a comparative metagenomic approach for functional characterization of multiple metagenome samples. J Microbiol Methods 115:121–128. doi:10.1016/j.mimet.2015.05.023 26027543

[60] Sunagawa S, Coelho LP, Chaffron S, Kultima JR, Labadie K, Salazar G, Djahanschiri B, Zeller G, Mende DR, Alberti A, et al.. 2015. Ocean plankton. Structure and function of the global ocean microbiome. Science 348:1261359. doi:10.1126/science.1261359 25999513

