# Supplementary material for: Analysis of the composition and function of rhizosphere microbial communities in plants with tobacco bacterial wilt disease and healthy plants
Source: Microbiol Spectr. 2024 Oct 29;12(12):e00559-24. doi: 10.1128/spectrum.00559-24 (PMC11622736; doi:10.1128/spectrum.00559-24)
Supplement: Supplemental figures — Fig. S1 to S3. [file spectrum.00559-24-s0001.pdf]

Supplemental figure

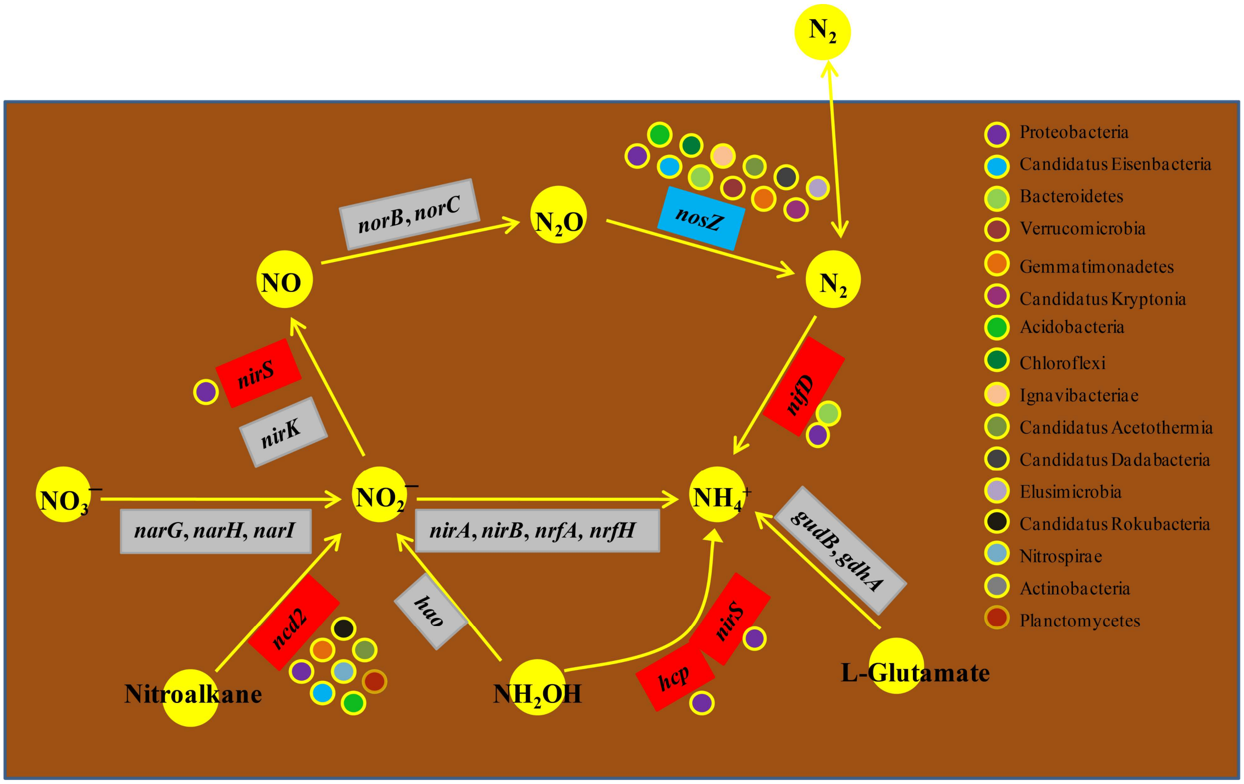

Fig. S1 Schematic representation of nitrogen cycling in healthy and diseased soils.

The blue box represents the high abundance level of genes in diseased soil, the red box represents the high abundance level of genes in healthy soil, the gray box represents the insignificant difference in gene abundance between two soils, and the colored circle represents the phylum where the gene is detected. APS = adenosine 5’-phosphosulfate.

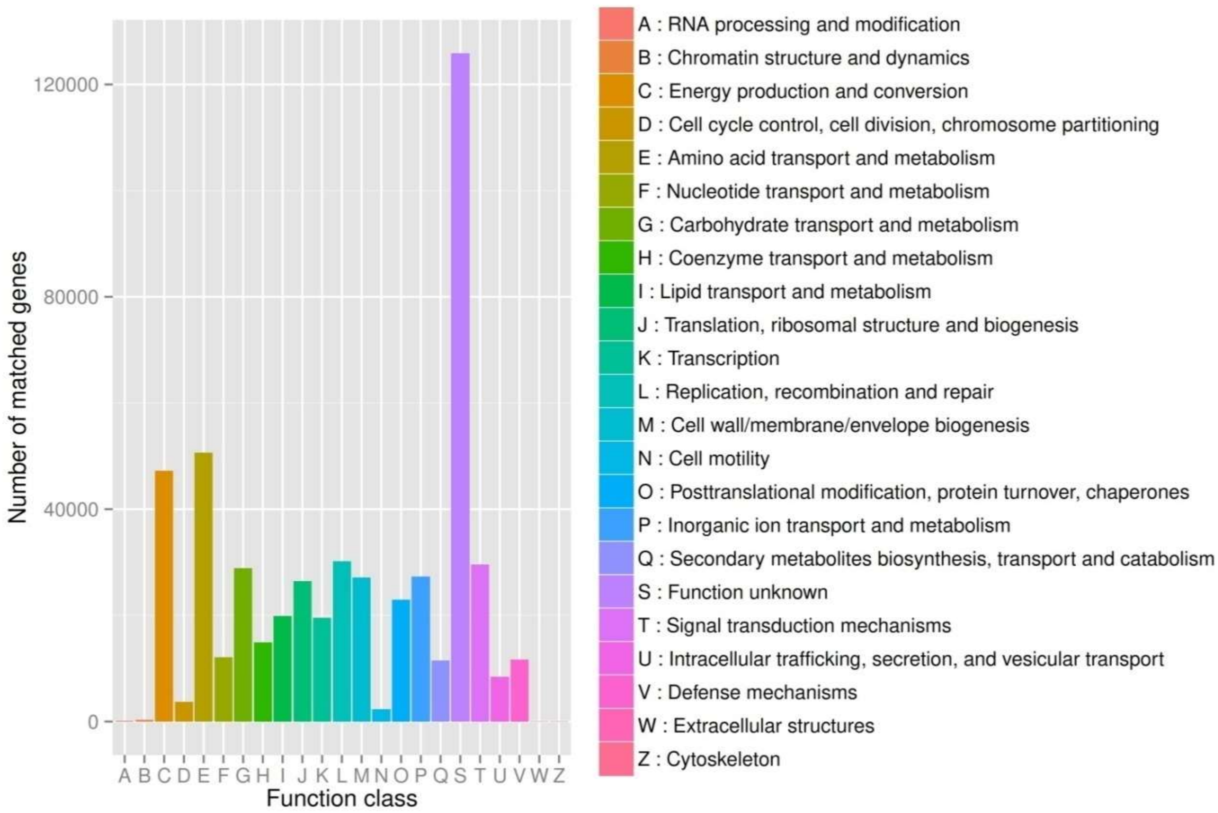

Fig. S2 Metagenomic sequencing to obtain eggNOG annotations for unigenes.

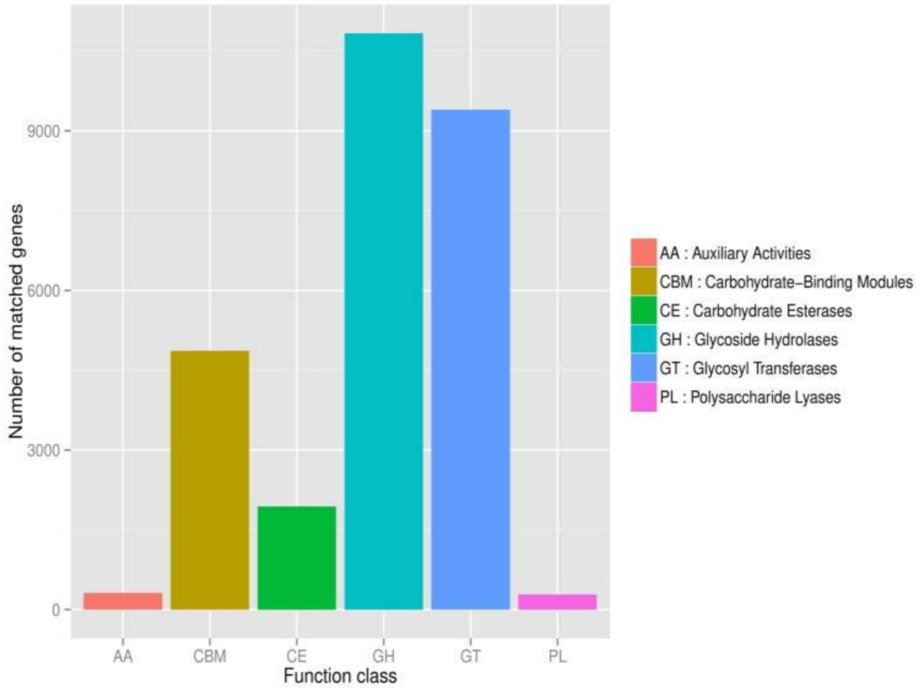

Fig. S3 CAZY annotation of unigenes obtained from metagenomic sequencing.
